# Editorial for the Special Issue on the ICAE 2019

**DOI:** 10.3390/mi11090874

**Published:** 2020-09-20

**Authors:** Hongsoo Choi, Dong-Weon Lee, Jeong-Bong Lee, Sang-Jae Kim

**Affiliations:** 1 Department of Robotics Engineering, Daegu Gyeongbuk Institute of Science & Technology, Daegu 42988, Korea; 2 School of Mechanical Engineering, Chonnam National University, Gwangju 61186, Korea; 3 Erik Jonsson School of Engineering and Computer Science, RL 10 The University of Texas at Dallas, 800 W. Campbell Rd., Richardson, TX 75080, USA; 4 Department of Mechatronics Engineering, Jeju National University, Jeju 63243, Korea

This special issue is a collection of 10 selected papers after presenting at the Fifth International Conference on Advanced Electromaterials (ICAE 2019), held in Jeju, South Korea on 5–8 November 2019. The ICAE is a bi-annual conference series hosted by The Korean Institute of Electrical and Electronic Material Engineers, focusing on all aspects of ‘electromaterials’. Its scope includes, but is not limited to, the various disciplines of electronic materials, including ferroelectric and piezoelectric materials, green energy materials such as solar cells and fuel cells, functional thin films and devices, energy storage materials, ‘soft’ organic electronics, and intelligent sensor materials and devices, new applications and displays based on light-emitting diodes (LEDs) and organic LEDs, advances in large-gap semiconductors, atomic and nano-scale characterization of electrical materials, and so on.

In this Special Issue, 10 technical papers are published on piezoelectric devices [1,2,3,4], biomedical devices [5,6], micromachining [7,8], and complementary metal-oxide-semiconductor (CMOS) logic devices [9,10]. For the piezoelectric devices, high-density piezoelectric micromachined ultrasonic transducer array based on patterned aluminum nitride thin film is introduced for imaging application [1]. A polyvinylidene fluoride-based composite matrix is presented for a complete polymer-based composite high-performance self-charging system [2]. Ring-type lead zirconate titanate ceramics prepared by the powder molding method are characterized for a high-intensity focused ultrasonic dispersion system [3]. Finally, the authors discuss the fundamental design of a hinge-lever type jet dispenser driven by a piezoelectric stacked actuator [4].

Two papers investigate polymer-based functional cantilevers integrated with interdigitated electrode arrays and magnetic microfluidic pump for novel cardiac sensing and in vivo bone remodeling application, respectively [5,6]. Reference [5] demonstrates a biomedical device that can simultaneously measure electrophysiology and contraction force of cardiomyocytes using a photosensitive polymer-based cantilever, with a pair of metal-based interdigitated electrodes on its surface. A magnetically operated implantable microfluidic pump is introduced for in vivo studies of bone intramedullary fluid modulation for the potential application in bone density augmentation study in rat femora [6].

A three-dimensional (3D) microlithography system with ultraviolet-LED (UV-LED) and low-temperature thin-film encapsulation using silicon nitride thin films are presented for the work on micromachining [7,8]. Computer-controlled UV-LED lithography was implemented for 3D microfabrication with an array of UV-LED and a tilt-rotational sample holder for 3D light traces [7]. Reference [8] investigates the role of an argon fluoride excimer laser beam with 193 nm wavelength in depositing silicon nitride thin films by laser-assisted plasma-enhanced chemical vapor deposition based on silane–ammonia gas mixtures toward encapsulation of polyethylene–naphthalate flexible substrates [8].

Finally, the effect of the deposition conditions for contact resistance in a multilevel CMOS logic interconnect device and process optimization through multilevel plug inter-connections in CMOS logic devices are discussed [9,10]. In the first paper, the authors present the optimal conditions in terms of the physical and electrical properties of the barrier and tungsten deposition process of a contact module, which is the segment that contains the device and the multi-layer metallization (MLM) metal line in the development of 100 nm-class CMOS logic devices [9]. In the second paper, they report on optimizing the device and wiring in a via structure applied to MLM used in CMOS logic devices [10].

After writing this editorial as the last part of this special issue, it was found that the electromaterials and micro-engineering have broad applications as discussed above, and they have the potential to develop new micro-engineered devices or to replace existing traditional devices. As many people are already using several micromachined-devices based on electromaterials and micro-engineering, the same trend will expand the applications in the next decade. Therefore, there will likely be more researchers in the fields of electromaterials and microtechnology, and many success stories are expected in the near future for the commercialization of the micro-engineered devices.

All editors of the special issue would like to thank all authors who have made a great contribution to its success. This special issue would not be possible without their contribution. We would also like to thank the reviewers for their time and effort in providing valuable feedback on each submitted paper. Our final thanks go to Ms. Mengdie Hu for her exuberance and support in completing this special edition.
